# Virtual reality gaming: a tool for reducing fear and anxiety in university students

**DOI:** 10.3389/fpsyg.2025.1532753

**Published:** 2025-04-23

**Authors:** Qian Zhang, Anlin Peng, Lihua He, Xincheng Li

**Affiliations:** ^1^Students’ Mental Health Education and Counseling Center, Guangxi University of Chinese Medicine, Nanning, Guangxi, China; ^2^Trade union in university, Guangxi University of Chinese Medicine, Nanning, Guangxi, China; ^3^Faculty of Orthopedics and Traumatology, Guangxi University of Chinese Medicine, Nanning, Guangxi, China; ^4^School of Public Health and Management, Department of Information Management and Information Systems, Guangxi University of Chinese Medicine, Nanning, Guangxi, China

**Keywords:** VR gaming, university students, fear, anxiety, mental health, empirical study

## Abstract

**Objective:**

This study aimed to investigate the effects of outdoor experiential training on the mental health of college students, specifically focusing on anxiety and fear, through the use of virtual reality (VR) technology.

**Methods:**

A total of 104 undergraduate participants (20 males and 35 females in both experimental and control groups) were recruited from our university. Participants, aged 18 to 23, were randomly assigned to an experimental group (*n* = 52) that utilized VR technology for emotional regulation training and a control group (*n* = 52) that underwent traditional relaxation training. All participants had good mental health status and provided written informed consent. The training lasted for 6 months, with 60-min sessions twice a week. Emotional states were assessed using the SCL-90 scale at baseline, mid-intervention, and post-intervention to evaluate the differences between groups.

**Results:**

Statistical analyses using SPSS 25.0 revealed significant reductions in anxiety and fear symptoms among participants in the experimental group compared to the control group. Specifically, anxiety scores decreased from an average rank of 5.36 to 4.59, and fear scores from 4.60 to 3.78 across the intervention phases. The total SCL-90 scores showed a significant improvement, decreasing from 6.39 to 5.41 (*p* < 0.05). These improvements were most pronounced during the second phase of the intervention, suggesting that repeated exposure to VR scenarios enhances emotional regulation and psychological resilience.

**Conclusion:**

The findings strongly indicate that VR technology is effective in alleviating anxiety and fear among college students, suggesting its potential as a viable intervention tool for mental health improvement in educational settings. The immersive nature of VR provides a safe and controlled environment for students to confront and manage their fears, which can be particularly beneficial for those who are reluctant to seek face-to-face counseling.

## 1 Introduction

### 1.1 Background

In recent years, the prevalence of anxiety and fear among university students has become a growing concern, with research indicating that over 30% of students report experiencing moderate to severe anxiety symptoms (Ahmed et al., 2023). These psychological issues not only impair academic performance and daily functioning but also pose long-term risks to students’ mental health, including increased susceptibility to depression and other psychiatric disorders (Zhang et al., 2020). The rising academic pressures, social challenges, and uncertainties about future career prospects have further exacerbated these emotional disturbances, making it imperative to develop effective interventions tailored to this population (Liu et al., 2022).

Traditional psychological interventions, such as cognitive behavioral therapy (CBT), have demonstrated efficacy in treating anxiety and fear (Olatunji et al., 2010; Reaven et al., 2012). However, their widespread adoption among university students is hindered by several barriers, including stigma, time constraints, and high costs (Aguirre Velasco et al., 2020). Many students are reluctant to seek professional help due to fears of being labeled as “mentally ill,” while others find it difficult to commit to the time-intensive nature of traditional therapies. These limitations highlight the need for alternative interventions that are more accessible, cost-effective, and less stigmatizing.

In virtual reality (VR), the virtual itself constitutes a genuine mode of reality, and “virtual reality” (Yoh, 2001) should be understood as “entities, agents, and events existing within cyberspace.” Virtual reality, hereinafter referred to as VR, as an emerging psychological intervention tool, offers new possibilities for addressing issues of anxiety and fear. Virtual reality (VR) has emerged as a promising tool in the field of psychological interventions, offering a novel approach to addressing anxiety and fear (Bell et al., 2024). Unlike traditional methods, VR creates immersive, controlled environments where individuals can safely confront and gradually overcome their fears (Scurati et al., 2021).

### 1.2 Research purpose

The primary objective of this study is to investigate the effectiveness of VR technology in reducing generalized anxiety and fear among university students, with a specific focus on high-altitude VR scenarios. By comparing pre-and post-intervention psychological health data, this research seeks to provide empirical evidence supporting the use of VR as a viable intervention tool in educational settings.

This study addresses a critical gap in the literature by exploring the potential of VR technology to alleviate generalized anxiety and fear among university students. While VRET has been well-documented for treating specific phobias, its application to broader psychological challenges faced by students remains underexplored (Cieślik et al., 2020). By focusing on high-altitude VR scenarios, this research contributes to both theoretical understanding and practical applications of VR in mental health interventions. Additionally, it highlights the importance of early intervention and the potential of VR to reduce stigma and improve accessibility to mental health support.

### 1.3 Research questions and hypotheses

To guide this study, the following research questions and hypotheses are proposed:

Research Question 1: Can VR-based gaming interventions effectively reduce anxiety levels among university students?

Hypothesis 1: Participants exposed to high-altitude VR gaming scenarios will exhibit a more significant reduction in anxiety scores compared to the control group, and the alleviation of anxiety symptoms will become more pronounced with increased intervention sessions.

Research Question 2: Can VR-based gaming interventions effectively reduce fear levels among university students?

Hypothesis 2: Participants exposed to high-altitude VR gaming scenarios will exhibit a more significant reduction in fear scores compared to the control group, and the alleviation of fear symptoms will become more pronounced with increased intervention sessions.

Research Question 3: What is the impact of VR-based gaming interventions on the overall mental health of university students?

Hypothesis 3: Repeated exposure to VR scenarios will lead to significant improvements in participants' overall mental health levels.

These research questions and hypotheses aim to systematically explore the effectiveness of VR-based gaming interventions in addressing anxiety, fear, and overall mental health among university students, providing a foundation for further empirical investigation and practical application.

## 2 Literature review

### 2.1 Psychological interventions for anxiety and fear

Traditional interventions for anxiety and fear include Cognitive Behavioral Therapy (CBT) (Hofmann et al., 2012) and exposure therapy (Neudeck and Wittchen, 2012). These approaches have achieved broad success by helping patients confront and gradually overcome their fears and anxieties (Rothbaum and Schwartz, 2002). However, research has also identified several limitations in these traditional methods, such as patient resistance, time constraints, and economic costs (DiMauro, 2014). Consequently, there is an urgent need to explore more flexible, cost-effective, and broadly applicable intervention methods.

### 2.2 Use of virtual reality technology

In recent years, virtual reality (VR) technology has emerged as an immersive tool for psychological interventions (Geraets et al., 2021). Virtual Reality Exposure Therapy (VRET) (Bush, 2008) utilizes simulated environments to allow individuals to gradually confront their sources of fear within a safe virtual context, thereby effectively alleviating symptoms of anxiety and specific phobias (Maples-Keller et al., 2017; Rothbaum et al., 2000). This technology addresses many limitations of traditional exposure therapy, such as the difficulty of replicating certain fear-inducing situations in real life (e.g., heights) (Flores et al., 2023).

### 2.3 The use of virtual reality technology in anxiety and fear

Research has demonstrated that virtual reality (VR) technology is highly effective in treating specific phobias, such as acrophobia, fear of flying, and social anxiety disorder. Virtual Reality Exposure Therapy (VRET) can safely and repeatedly recreate fear-inducing scenarios within a controlled laboratory environment, allowing patients to gradually acclimate to these fears within a secure virtual context (Wiederhold et al., 2014).

Rimer et al. demonstrated that Virtual Reality Exposure Therapy (VRET) is highly effective in treating acrophobia, with patients showing significant reductions in symptoms following multiple exposures to virtual high-altitude scenarios. These findings provide robust evidence for the efficacy of virtual reality technology in phobia interventions (Rimer et al., 2021).

### 2.4 Intervention of anxiety and fear in college students using virtual reality technology

In the university student population, anxiety and fear are prevalent due to academic pressure, social stress, and uncertainty about the future (Zhang et al., 2018). Surveys indicate that over 30% of university students report moderate to severe anxiety symptoms, which have a significant negative impact on their academic performance and daily life (Jia et al., 2023).

Among Chinese university students, anxiety and fear primarily arise from academic pressure (Zhang et al., 2020). This pressure is often manifested in concerns related to exam performance, academic workload, and future employment prospects. For instance, academic performance is closely tied to future job opportunities, leading students to place significant emphasis on their exam results.

Additionally, Chinese university students frequently experience anxiety due to concerns about exam results and course difficulty. This anxiety often stems from worries about an uncertain future and manifests as excessive worry, test-related nervousness, and difficulty concentrating on their studies (Wu et al., 2020).

These emotional disturbances are often temporary and typically associated with the end of a semester, exam periods, or specific academic tasks. When the sources of stress are removed, students’ anxiety and fear generally diminish or disappear. Although these emotions may impact students’ daily lives and academic performance, they usually do not reach the severity required for a clinical diagnosis. Such mild emotional disturbances typically do not necessitate professional psychological treatment from hospitals but may require universities to provide students with coping strategies and support.

Current research primarily focuses on anxiety or fear in specific patient populations, with relatively few comprehensive studies addressing the mild anxiety and fear experienced by the majority of university students.

Traditional psychological interventions in Chinese universities, such as psychological counseling and group therapy, although effective, are often challenging to implement widely within this population. Particularly under high academic pressure, Chinese university students frequently find it difficult to engage in ongoing psychological interventions. Therefore, identifying more appealing and applicable intervention methods is crucial for addressing this issue (Huang et al., 2018).

## 3 Methods

### 3.1 Participants

To investigate the impact of virtual reality (VR) games on reducing fear and anxiety among college students, we recruited undergraduate students from Guangxi University of Chinese Medicine. Participants were recruited through campus posters, the official WeChat account of Guangxi University of Chinese Medicine, and QQ groups of various undergraduate classes. The recruitment advertisement clearly stated the purpose of the study, the intervention content, and the rights and obligations of the participants. All participants were undergraduate students from Guangxi University of Chinese Medicine. Initially, a total of 110 participants enrolled, with an age range of 18 to 23 years (mean age = 19.33 ± 0.94 years). Each group consisted of 20 males and 35 females. Participants were from different grades and majors to ensure sample diversity.

The inclusion criteria for participants included: (1) good mental health confirmed by preliminary screening with the Symptom Checklist-90 (SCL-90); (2) no history of mental illness; (3) no physiological conditions that may pose a risk to VR training (such as asthma or heart disease). All participants signed a written informed consent after fully understanding the purpose, procedures, and potential risks of the study.

However, since participation was voluntary, there may be a self-selection bias, meaning that students who were more interested in or motivated to participate in mental health interventions were more likely to enroll. This bias may limit the generalizability of the study findings to other populations. Based on an anticipated attrition rate of 5.5%, a total of 104 participants completed the study (52 in the experimental group and 52 in the control group).

**Figure 1:**
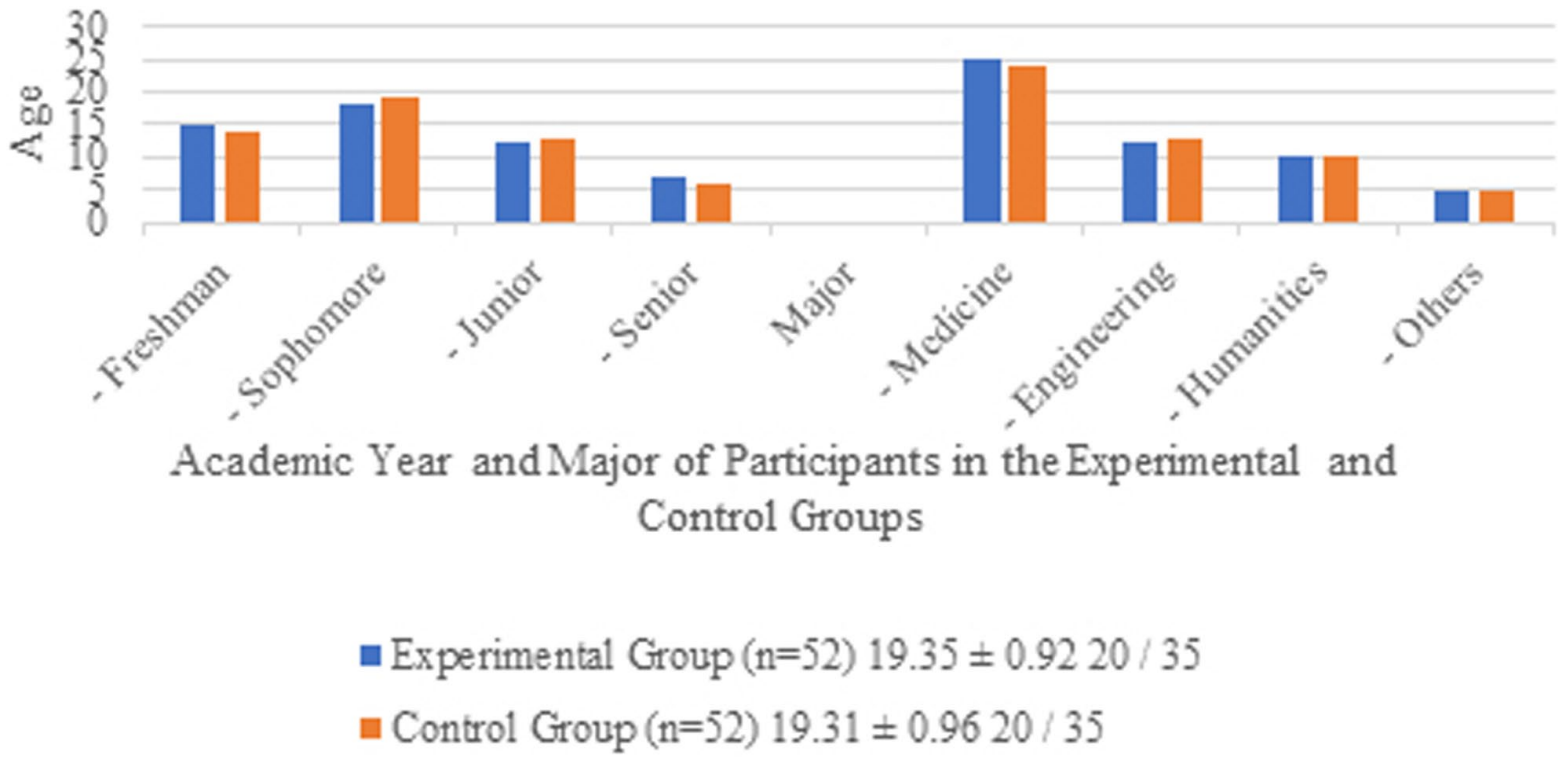
Demographic of participants in the experimental and control groups.

The following is a table of the demographic data of the participants:

Table 1 and Figure 1 present the demographic and academic characteristics of participants in the experimental and control groups. The sample consisted of 104 participants, evenly divided into two groups (*n* = 52 each). The mean age of participants was approximately 19.3 years in both groups, with a standard deviation of less than 1 year, indicating a homogeneous age distribution. Gender distribution was identical across groups, with 20 males and 35 females in each. In terms of academic year, the majority of participants were freshmen and sophomores, with fewer juniors and seniors. Regarding majors, medicine was the most common field of study, followed by engineering, humanities, and others. The data suggest that the two groups were well-balanced in terms of demographic and academic variables, which supports the validity of the comparative analysis.

**Table 1:** Demographic of participants in the experimental and control groups.

Characteristic	Experimental group (*n* = 52)	Control group (*n* = 52)
Age (mean ± SD)	19.35 ± 0.92	19.31 ± 0.96
Gender (male/female)	20/35	20/35
Academic year		
Freshman	15	14
Sophomore	18	19
Junior	12	13
Senior	7	6
Major		
Medicine	25	24
Engineering	12	13
Humanities	10	10
Others	5	5

### 3.2 Experimental objectives

The primary objective of this study is to investigate the effectiveness of VR-based gaming interventions in reducing anxiety and fear among university students. Specifically, the study aims to:

1. Evaluate the effectiveness of VR games in reducing participants’ fear symptoms, as measured by the SCL-90 scale.
2. Evaluate the effectiveness of VR games in reducing participants’ fear symptoms, as measured by the SCL-90 scale.
3. To evaluate the effectiveness of VR gaming in improving the overall health status of the participants, as measured by the SCL-90 scale.

By achieving these objectives, this study seeks to provide empirical evidence supporting the use of VR as a viable intervention tool for mental health improvement among university students.

### 3.3 Experimental tasks

#### 3.3.1 VR training protocol

The experimental group participated in a 6-month VR training program, with two 60-min sessions per week. The VR training utilized the Oculus Quest 2 headset, preloaded with a high-altitude climbing game (The Climb 2). The game included five maps, each with three difficulty levels (beginner, intermediate, and advanced). Participants could choose between Practice Mode (offline, no time constraints) and Competition Mode (online, time-limited). The training focused on gradually exposing participants to fear-inducing scenarios (e.g., climbing mountains and skyscrapers) to help them manage anxiety and fear.

#### 3.3.2 Justification for scenario selection

The selection of mountain climbing and skyscraper scenarios was based on their relevance to common fears experienced by university students, particularly those related to heights and spatial challenges. These scenarios were chosen because they provide a controlled yet immersive environment for participants to confront and gradually overcome their fears. Additionally, the high-altitude settings are universally recognized as fear-inducing, making them suitable for addressing generalized anxiety and fear, which are prevalent among students due to academic and social pressures. The gradual progression from beginner to advanced levels ensures that participants can build confidence and emotional resilience over time.

#### 3.3.3 Control group protocol

Participants in the control group did not receive any VR intervention or additional psychological training. They were instructed to continue their normal daily routines and were assessed at the same time points as the experimental group.

#### 3.3.4 Randomization

Participants were randomly assigned to the experimental or control group using Microsoft Excel. A random number generator was used to ensure unbiased allocation. The first 52 participants in the randomized list were assigned to the experimental group, and the remaining 52 were assigned to the control group.

### 3.4 Measurement tools

The Symptom Checklist-90 (SCL-90) is a widely used self-report instrument designed to evaluate a broad range of psychological problems and symptoms of psychopathology. It encompasses nine primary symptom dimensions: somatization, obsessive-compulsive, interpersonal sensitivity, depression, anxiety, hostility, phobic anxiety, paranoid ideation, and psychoticism. Additionally, it includes a global severity index to provide an overall measure of distress. The SCL-90 is utilized in both clinical and research settings to assess the psychological status of individuals and to monitor changes over time or in response to treatment (Müller et al., 2010).

The primary outcome measurement tool employed in this study was the Symptom Checklist-90 (SCL-90), hereinafter referred to as SCL-90. We selected the SCL-90 due to its high reliability (Cronbach’s alpha = 0.967) and its extensive application in mental health research (Derogatis and Unger, 2010). Participants completed the SCL-90 at three time points: baseline (pre-intervention), mid-intervention (3 months), and post-intervention (6 months).

#### 3.4.1 Secondary measures

To complement the SCL-90, physiological measures such as heart rate variability (HRV) were recorded during VR sessions to assess participants’ stress responses. However, these data were not included in the current analysis due to technical limitations.

### 3.5 Experimental procedures

We have developed a research schedule in Table 2. This study adopts a randomized controlled trial design. The experimental group receives VR-based emotion regulation training, and the control group maintains daily activities. The study will start in November 2023 and last until October 2024. It is divided into the preparation phase, equipment preparation, experimental process setting, three SCL-90 tests, two VR trainings, data collection and collation, data analysis and conclusion, and paper writing. The specific time schedule Table 2 is as follows.

**Table 2:** Research timeline.

Time period	Phase	Activity description
November 2023	Preparation phase	Recruit researchers and participants, finalize the research plan.
December 2023	Equipment setup	Purchase and rent VR equipment and training materials; ensure devices are properly configured.
January 2024	Experimental setup	Establish the experimental protocol: two sessions per week, 60 min each; randomize participants into experimental and control groups.
January 2024	First SCL-90 test	Conduct baseline assessment (SCL-90) to record pre-intervention anxiety, fear, and overall mental health data.
February–May 2024	First VR training	Experimental group undergoes the first phase of VR game training; control group maintains daily activities.
May 2024	Second SCL-90 test	Conduct mid-term assessment (SCL-90) to record anxiety, fear, and overall mental health data during the intervention.
May–August 2024	Second VR training	Experimental group undergoes the second phase of VR game training; control group maintains daily activities.
August 2024	Third SCL-90 test	Conduct post-intervention assessment (SCL-90) to record anxiety, fear, and overall mental health data after the intervention.
August 2024	Data collection and organization	Organize all experimental data and perform preliminary analysis.
September 2024	Data analysis and conclusion	Analyze the data and draw research conclusions.
October 2024	Paper writing	Write the research paper, summarize findings, and propose recommendations.

According to the research timeline in Table 2, we developed a flowchart of the steps of the research (including experiments), as follows.

**Figure 2:**
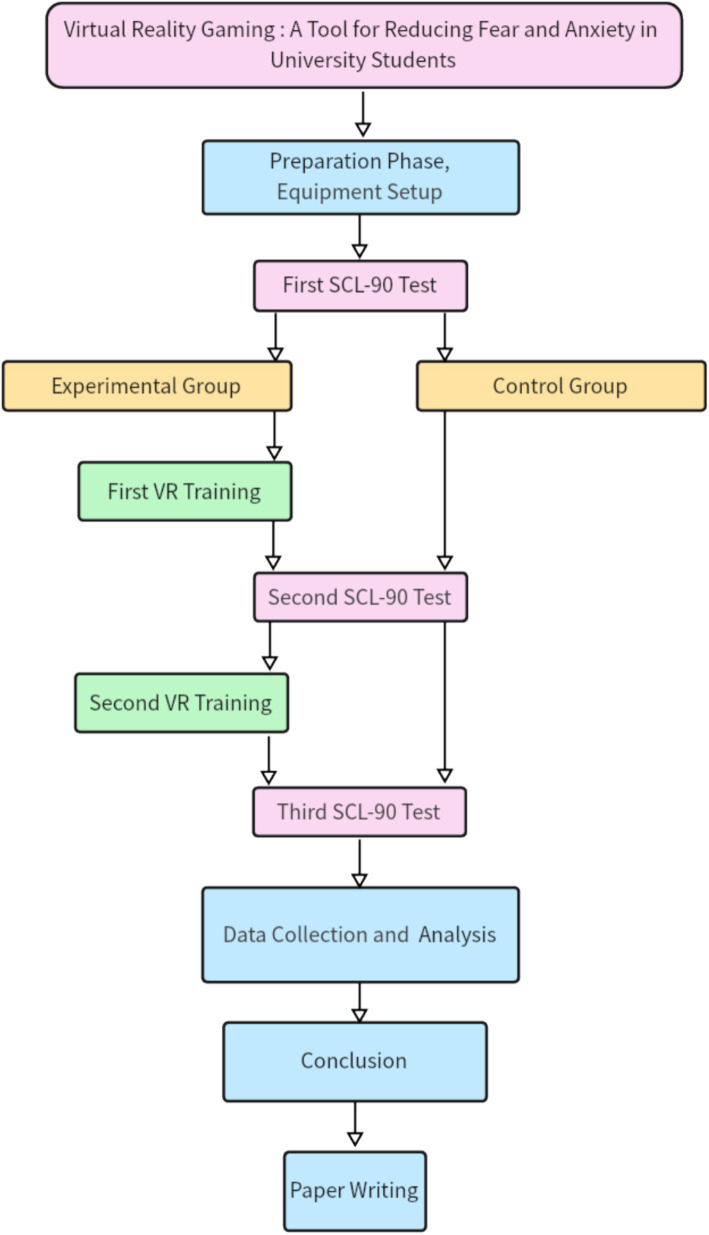
Flowchart of the effectiveness of VR games in reducing anxiety, fear, and overall psychological health among college students.

Figure 2 illustrates the experimental design and workflow of the study, which aims to evaluate the effectiveness of virtual reality (VR) games in reducing anxiety, fear, and overall mental health issues among college students. The study employs a randomized controlled trial (RCT) design, with participants divided into an experimental group and a control group.

The contents of each block in the flowchart are explained as follows:

#### 3.5.1 First SCL-90 test

At the baseline, all participants completed the Symptom Checklist-90 (SCL-90) to assess their initial levels of anxiety, fear, and overall mental health. This step ensured that both groups were comparable before the intervention.

#### 3.5.2 First VR training

Experimental Group: Participants in the experimental group engaged in the first stage of VR gaming using VR glasses. The VR game involved high-altitude climbing scenarios designed to simulate fear-inducing environments.

##### 3.5.2.1 Control group

Participants in the control group underwent the first stage of meditation, a traditional relaxation technique, as a non-VR intervention.

#### 3.5.3 Second SCL-90 test

After the first practice session, both groups completed the SCL-90 again to measure any immediate changes in anxiety, fear, and overall mental health.

#### 3.5.4 Second VR training

##### 3.5.4.1 Experimental group

Participants in the experimental group engaged in the second stage of VR gaming, which involved more challenging high-altitude scenarios to further test their emotional regulation abilities.

##### 3.5.4.2 Control group

Participants in the control group underwent the second stage of meditation, continuing the traditional relaxation training.

#### 3.5.5 Third SCL-90 test

After the second practice session, both groups completed the SCL-90 for the final time to assess the cumulative effects of the interventions.

#### 3.5.6 Data collection and analysis

The data collected from the three SCL-90 assessments were analyzed to compare the changes in anxiety, fear, and overall mental health between the experimental and control groups. Statistical analyses included descriptive statistics, non-parametric tests (Friedman test), and effect size calculations.

The flowchart provides a clear and concise overview of the study’s methodology, highlighting the key steps and interventions used to evaluate the impact of VR technology on mental health outcomes. The use of both experimental and control groups, along with repeated measures of the SCL-90, ensures the robustness and validity of the findings.

## 4 Conclusion

Based on the data analysis, the results were obtained regarding the effectiveness of VR games in reducing anxiety and fear among college students. The findings were explained in the context of the research hypotheses and research questions.

### 4.1 Paper writing

Based on the data and conclusions obtained from this study, write the paper and prepare it for submission.

### 4.2 Statistical analysis

Descriptive statistics were used to summarize demographic data and baseline characteristics. Non-parametric tests (Friedman test) were employed to analyze changes in SCL-90 scores over time, as the data did not meet the assumptions of normality. Effect sizes and confidence intervals were calculated to assess the magnitude of the intervention’s impact.

### 4.3 Experimental tools and steps

#### 4.3.1 VR glasses experimental procedures

**Figure 3:**
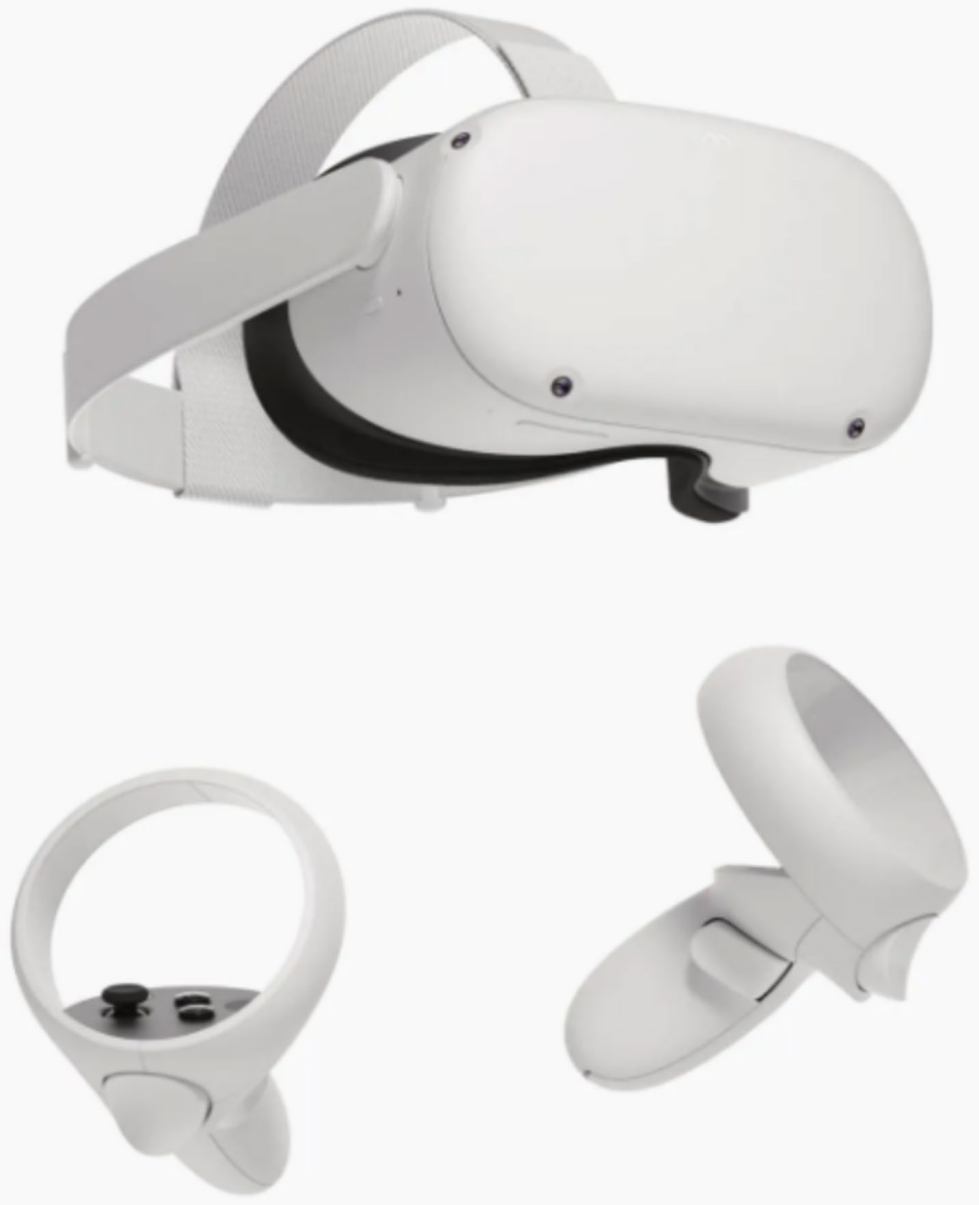
Quest 2 VR glasses device.

Figure 3 illustrates the Meta Quest 2, a standalone VR headset that provides wireless, untethered VR experiences without external sensors.

The Quest 2 devices used in the experiment included a VR headset, a charging cable and power adapter, two Touch controllers with accompanying AA batteries, and a glasses spacer. Prior to the experiment, all devices were rigorously inspected, cleaned, and tested to ensure proper functionality. The devices were preloaded with a high-altitude climbing game relevant to the experimental procedures and equipped with 128GB of storage capacity, providing a stable operational environment for the study.

In the Oculus Quest 3 standalone virtual reality (VR) headset system, there is a game called The Climb 2. This game includes five maps, each representing a different difficulty level for participants to choose from. Each map is further divided into three difficulty levels. After selecting a difficulty level, participants can choose between Practice Mode and Competition Mode. Practice Mode can be completed offline, whereas Competition Mode requires an internet connection and is more challenging compared to Practice Mode.

During the climbing process, Practice Mode does not include a stamina bar, allowing participants to hold onto a point on the mountain indefinitely. In contrast, Competition Mode includes a stamina bar, limiting the time a participant can hold onto a spot. An energy ring on the wrist decreases over time; once depleted, the participant loses their grip and falls in the virtual environment. At that point, they may restart the game.

Each difficulty level contains three distinct maps, with increasing levels of challenge. Reaching a platform counts as the completion of a stage. In the first experimental session, participants were instructed to select the “Mountain Climbing” option in *High Altitude Climbing 2*. After completing the “Mountain Climbing” task in the first session, participants were guided to choose the more challenging “City Building Climbing” option in the second session.

By the end of the first experimental session, all participants completed the beginner level of “Mountain Climbing” in Practice Mode. The following image shows the route map for the “Mountain Climbing” game.

**Figure 4:**
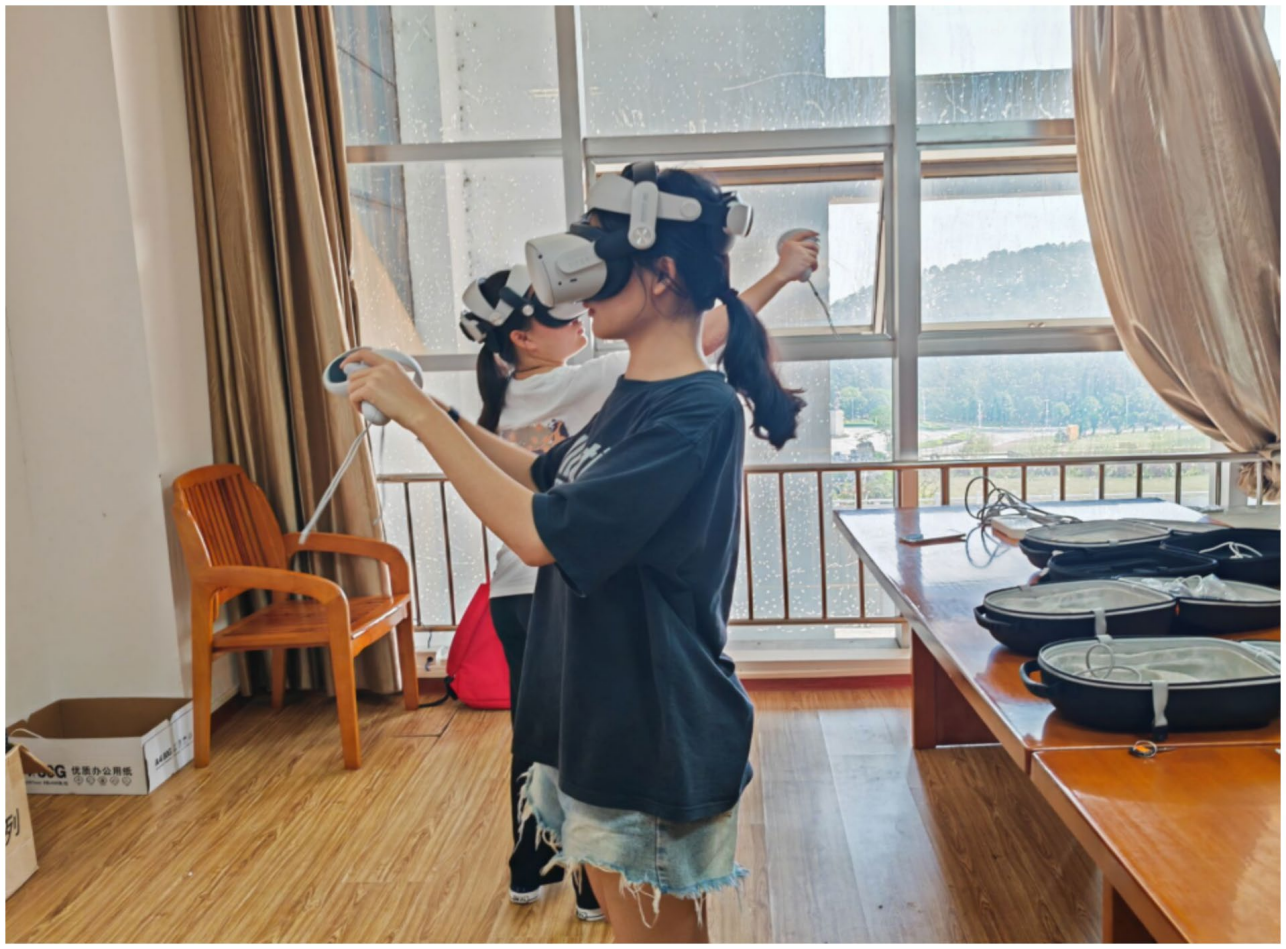
University students practicing with VR glasses.

Figure 4 illustrates the experimental group’s participants using VR headset equipment in a laboratory setting. A dedicated room was arranged for VR headset training specifically for the experimental group. Participants scheduled their practice sessions in advance with research assistants and entered the training room at their designated times. During the training, research assistants and project instructors provided guidance to ensure proper use of the VR headset and associated programs. Participants were allowed to contact the research assistants or project instructors at any time to address issues or restart their VR training sessions if necessary.

**Figure 5:**
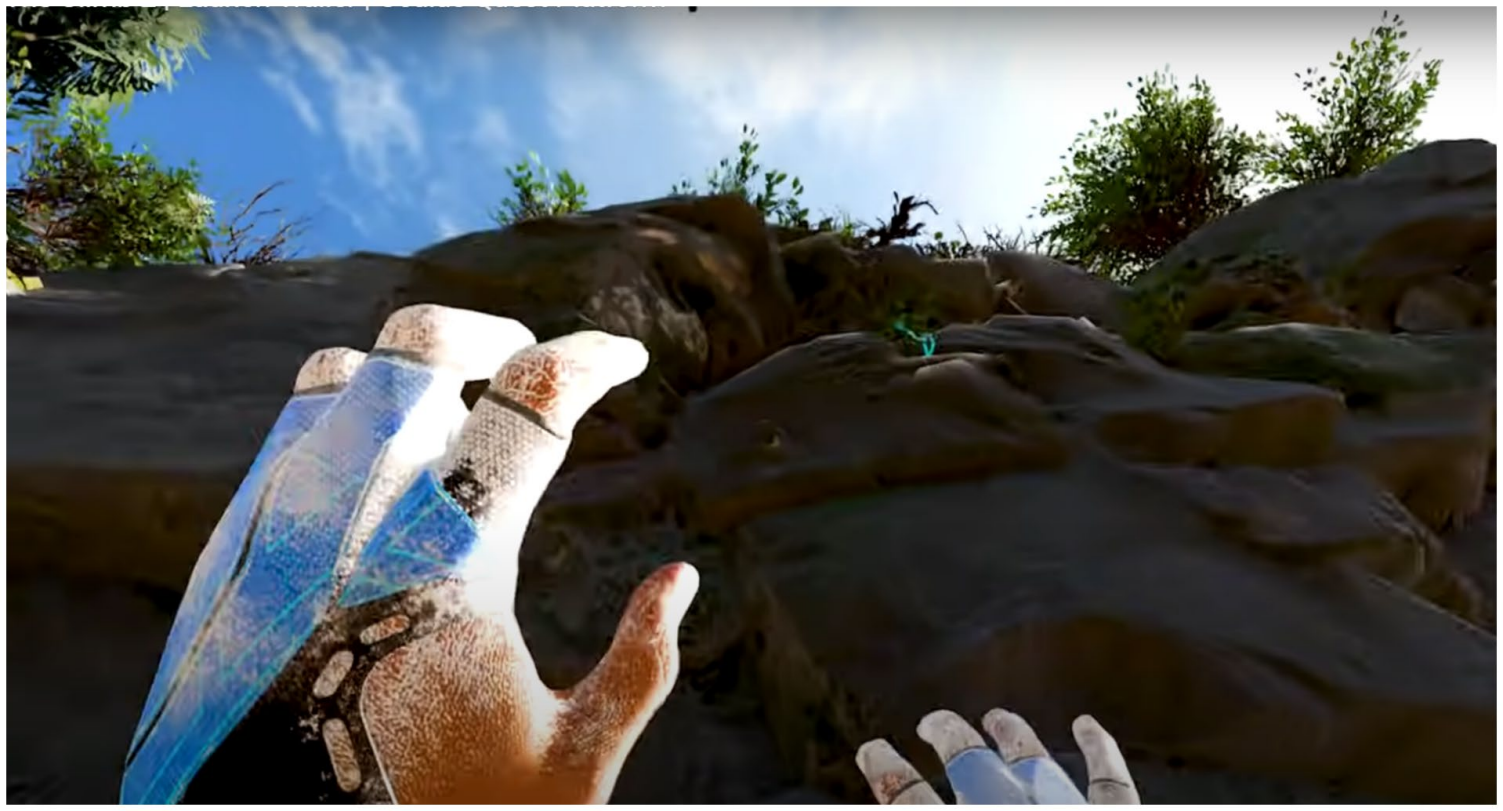
The interface seen by university students while alpine rock climbing in VR glasses.

Figure 5 illustrates the virtual interface during the first stage of the mountain climbing task for the experimental group. Participants began at the base of the mountain and progressively climbed toward the midsection. The virtual environment offered multiple climbing routes to choose from, requiring participants to locate handholds and use alternating hand grips to ascend, ultimately reaching the summit. The background, featuring mountainous and forest landscapes, created a realistic high-altitude environment for the experiment. During the early stages of practice, participants needed several attempts to adapt to the virtual climbing environment and master the operational techniques. The results indicate that most participants, after multiple failed attempts, were eventually able to complete the game objective of reaching the summit.

**Figure 6:**
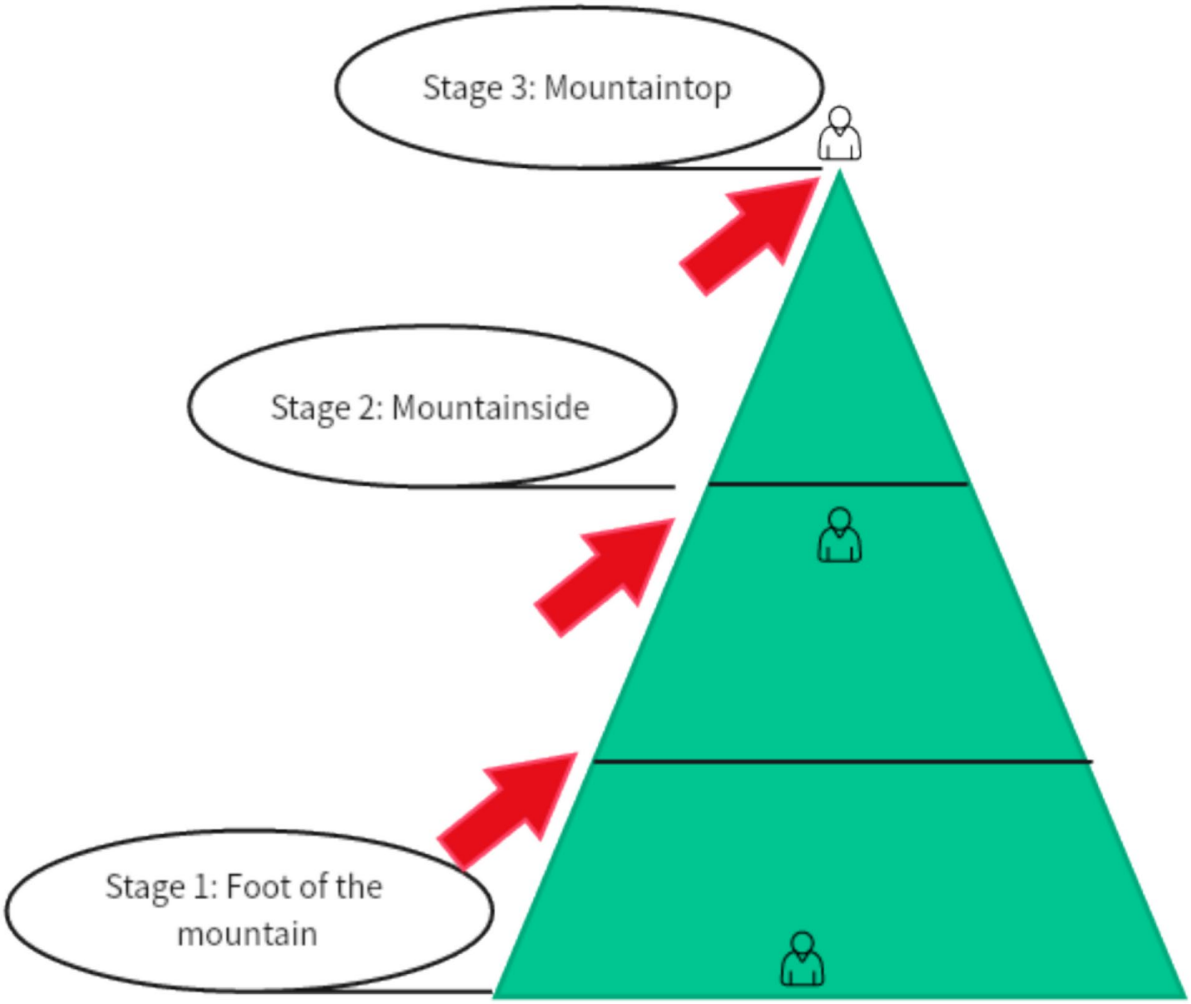
Steps of alpine climbing in VR glasses.

Figure 6 illustrates the climbing routes taken by the experimental group participants during the mountain climbing task. Participants started at the base of the mountain and climbed progressively toward the midsection. Multiple routes were available, requiring participants to locate handhold points and use alternating hand grips to ascend along the designated points, ultimately reaching the summit. During the initial practice sessions, participants required several attempts to adapt to the virtual climbing environment and mechanics.

**Figure 7:**
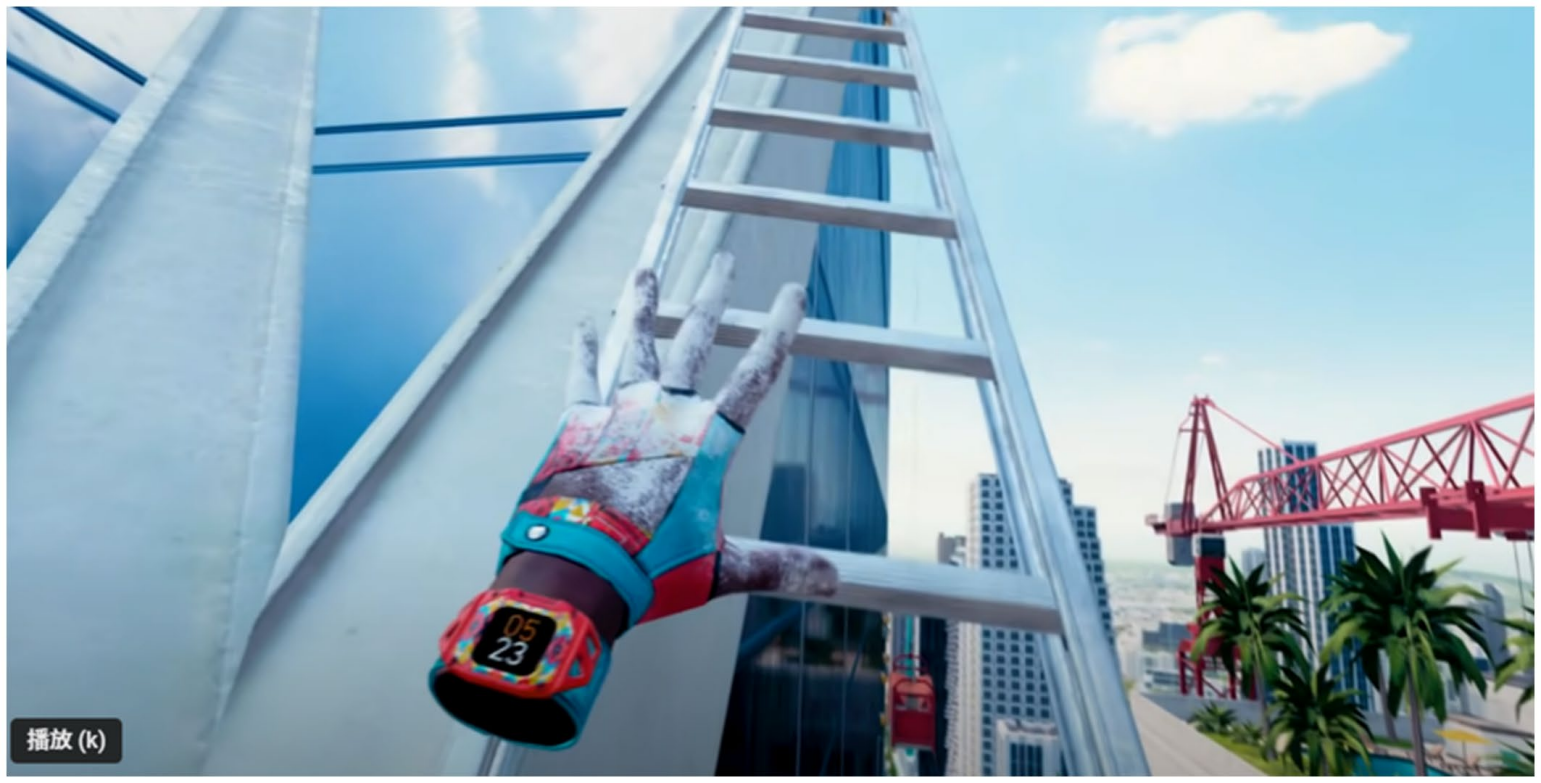
The interface seen by college students while climbing a city building in VR glasses.

Figure 7 depicts a segment of the virtual interface during the second stage of the high-altitude climbing task for the experimental group. Participants used virtual controllers to locate climbing points (e.g., ladders or protruding structures on skyscrapers) and simulated alternating hand grips to ascend. The background, featuring skyscrapers and urban landscapes, provided a highly realistic high-altitude environment, significantly enhancing the immersive experience. In the early stages of the game, participants required time to adapt to the virtual environment and gradually master proper climbing techniques.

**Figure 8:**
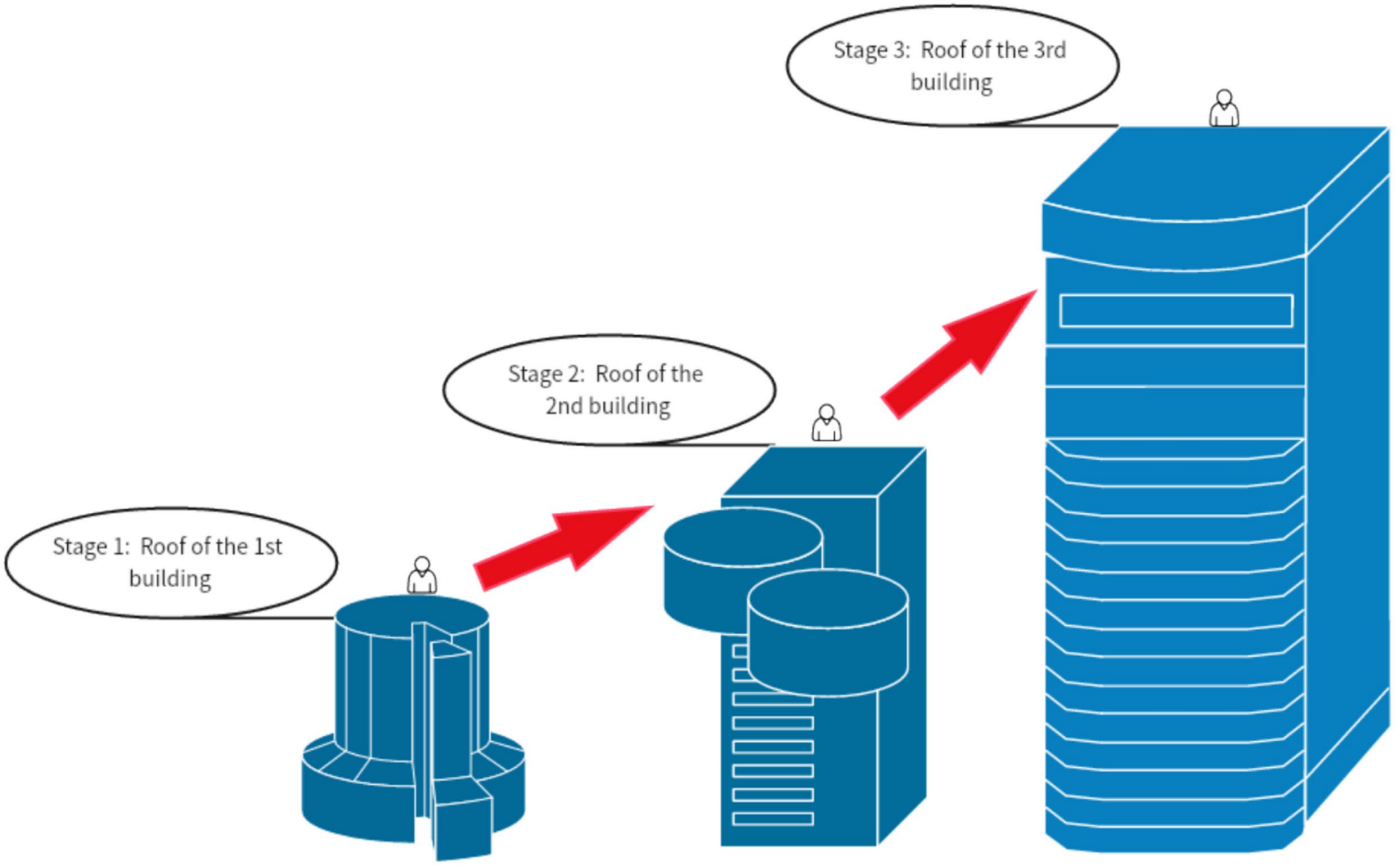
Steps for climbing a city building in VR glasses.

Figure 8 illustrates the climbing route in the second stage of the urban high-rise climbing task for the experimental group. In this task, participants start at the base of the first building and climb to the roof, then ascend to the roof of the second building, and finally reach the roof of the third building. The virtual environment offers multiple climbing paths, requiring participants to locate suitable handholds and use alternating hand grips to ascend. The design of the task enhances the challenge with multiple climbing stages, requiring participants to gradually adapt to different levels of climbing difficulty. During the early stages, experimental group members needed several attempts to adapt to the virtual high-rise environment and master the proper climbing techniques.

#### 4.3.2 The control group did not receive VR glasses intervention

In this study, participants in the control group will not receive any intervention. During the experiment, control group participants will maintain their usual daily activities, without engaging in any form of psychological intervention, meditation training, or other relaxation exercises. The main purpose of the control group is to serve as a baseline for comparison with the experimental group, which will receive virtual reality (VR) intervention. This design allows the study to evaluate the effectiveness and impact of VR intervention in reducing anxiety and fear among university students.

### 4.4 Measurement tool

The SCL-90 is a comprehensive self-report questionnaire used to assess a range of psychological symptoms and distress. The scale includes 90 items covering nine symptom dimensions: somatization, obsessive-compulsive symptoms, interpersonal sensitivity, depression, anxiety, hostility, phobic anxiety, paranoid ideation, and psychoticism. Participants rate each item on a 5-point Likert scale (0 = Not at all, 1 = A little, 2 = Moderately, 3 = Quite a bit, 4 = Extremely). The scores for each dimension are obtained by summing the ratings of all items, and a Global Severity Index is also calculated. Higher scores indicate more severe symptoms (Derogatis and Unger, 2010; Zhou et al., 2021).

This study uses the Symptom Checklist-90 (SCL-90) to assess participants’ emotional states and mental health levels before, during, and after the experiment. Data analysis will be conducted using the statistical software SPSS 25 to compare the differences in scale scores between the experimental group and the control group, in order to evaluate the effectiveness of virtual reality technology in emotion regulation training. Based on the data analysis results, the study will interpret and discuss the experimental findings and propose recommendations and directions for further research.

### 4.5 Experimental procedure

The overall research framework for the application study of using virtual reality technology for emotional regulation training in college students can be divided into the following steps:

#### 4.5.1 Step 1: design the experimental and control groups

The experimental group (*n* = 50): Conduct emotional regulation training using virtual reality (VR) technology. Each session includes an immersive experience in a virtual high-altitude scenario, combined with breathing regulation and anxiety management techniques.

The control group (*n* = 50): Use traditional emotional regulation methods, such as breathing relaxation training, without the use of VR technology.

We provided four weekly time slots for experimental group participants to access the laboratory. Participants could select and schedule two practice sessions per week by filling out their availability online. Researchers collected this information and arranged the specific open times accordingly. The laboratory was a spacious area with minimal furnishings, featuring only desks and chairs placed along the walls and an air conditioning system, ensuring a distraction-free environment for the experiments.

#### 4.5.2 Step 2: pre-experiment psychological scale assessment

Before the start of the experiment, all participants completed the Symptom Checklist-90 (SCL-90), to obtain baseline emotional state data.

#### 4.5.3 Step 3: emotional regulation training in a virtual reality setting

The experimental period lasted for six months, divided into two phases, each lasting three months. Participants in the experimental group underwent VR-based training sessions twice a week, with each session lasting 60 min. The VR technology simulated high-altitude scenarios to facilitate emotion regulation practices. In contrast, the control group did not receive any VR intervention. A training interval of 2–3 days was maintained between sessions to ensure participants had sufficient time for emotional recovery and to avoid overexposure or fatigue.

#### 4.5.4 Step 4: mid-experiment psychological scale assessment

At the mid-point of the experiment (after 3 months), the experimental and control groups will again complete the psychological health scales to assess changes in emotional state during the training period.

#### 4.5.5 Step 5: psychological scale test at the end of the experiment

At the end of the experiment (at the 6-month mark), all participants will complete the SCL-90, SDS, and SAS scales again to assess the overall intervention effects of the training on emotional and mental health.

#### 4.5.6 Step 6: data analysis

We used SPSS 25.0 software to analyze the data collected at three time points: pre-experiment, mid-experiment, and post-experiment. The analysis focused on evaluating the effects of virtual reality-based emotion regulation training and comparing differences between the experimental and control groups. Statistical methods included descriptive analysis, difference analysis, correlation analysis, and reliability and validity testing of the scales. Additionally, regression analysis was performed to assess changes in emotional states within and between the experimental and control groups before and after the intervention, providing a comprehensive evaluation of the effectiveness of the VR intervention.

#### 4.5.7 Step 7: summarize research findings

Based on the data analysis results, discuss the impact of virtual reality technology on emotional regulation abilities and mental health. Additionally, propose directions for further research and improvement suggestions in light of the study’s findings.

### 4.6 Experimental tools

The experimental group will use virtual reality (VR glasses) for scenario simulation and emotional regulation training. Research assistants will guide participants in selecting the virtual climbing game and virtual public speaking game within the VR glasses for training. The virtual climbing game will include types of climbing scenarios, such as mountains and tall buildings. During the virtual climbing game, participants will see scenes through the VR glasses that resemble those encountered in real rock climbing, such as mountains, forests, rocks, etc. Participants will not encounter sudden appearances of giant animals or unexpected frightening images. The visual experience for participants is shown in the figure below:

The theoretical basis of this study is exposure therapy, a widely used psychological treatment method to improve emotional well-being, primarily for treating anxiety disorders, post-traumatic stress disorder (PTSD), obsessive-compulsive disorder (OCD), and other conditions. It involves gradually exposing patients to scenarios or memories related to their fears, anxieties, or traumas in a safe environment to help them gradually reduce their fear and avoidance of these stimuli.

In exposure therapy, virtual reality technology can be used to simulate these fear or trauma-related scenarios, such as heights or public places. Participants can experience these simulations through virtual reality technology to gain a more realistic experience, thereby better managing their fears or avoidance.

### 4.7 Experimental training method

Training Method of This Project – Immersion in Virtual Fear Scenarios:

Fear virtual scenarios are tools used to help individuals overcome their fear symptoms. The following are the steps for setting up fear virtual scenarios:

1. Identify the Fear Object: Heights (e.g., climbing).
2. Create Virtual Scenes: Use virtual reality technology or computer simulation software to create a virtual scenario. Initially, design a virtual mountain for climbing, and then progress to a virtual high-rise building.
3. Set Scene Details: Include environmental sounds, visual effects, and the appearance of fear objects to make the virtual scenario more realistic.
4. Guide Users into the Virtual Scenario: Allow users to feel as though they are within the virtual environment.
5. Adjust Scenario Intensity: Based on users’ reactions, gradually adjust the intensity of the scenario to make it increasingly similar to real-life situations.
6. Apply Gradual Exposure: Use a progressive exposure method within the virtual scenario, gradually exposing users to more intense fear scenarios until they adapt to the fear object.

Through training in virtual scenarios, users can progressively confront and overcome their anxiety and fear, thereby improving their psychological well-being.

### 4.8 Criteria for termination of the study

This study will strictly adhere to ethical standards to ensure the safety and rights of participants. The following are the explicit criteria for terminating participation in this study:

1. Voluntary Withdrawal: Participants have the right to withdraw from the study at any time without providing a reason. Upon withdrawal, all data related to the participant will be destroyed and will no longer be used for research analysis.
2. Adverse Reactions: If participants experience severe dizziness, headaches, worsening anxiety, or other adverse reactions during virtual reality training, their participation will be immediately terminated. Appropriate measures will be taken as outlined below.
3. Non-compliance: If participants repeatedly fail to attend training sessions on time or do not complete measurements as required, researchers will communicate with the participant and may terminate their participation based on the situation.
4. Potential Harm: After a comprehensive assessment of participants’ performance and reactions, if researchers believe that continued participation may cause harm, the decision to terminate participation will be made, and the participant will be promptly notified.
5. Inquiries: Participants may contact the research team at any time if they have questions or need further clarification.

#### 4.8.1 Measures for adverse psychological states

If participants exhibit adverse psychological states during the study, the following measures will be taken to ensure their mental health and provide necessary support and assistance, avoiding any form of psychological harm:

1. Initial Assessment: Participants displaying adverse psychological states will be assessed by a professional psychologist from the research team to determine the severity of the condition.
2. Initial Support: In the event of initial adverse psychological states, the research team will immediately provide psychological support, including listening, comforting, and preliminary psychological intervention. If initial interventions are ineffective or the participant’s condition is severe, a one-on-one consultation with a professional psychologist will be arranged.
3. Referral to Professional Care: If the psychological issues exceed the research team’s capability to handle, participation in the experiment will be terminated to ensure the participant is no longer involved in research activities. The participant will be referred to a professional hospital for comprehensive treatment. During the referral process, the research team will assist the participant and their family, providing necessary support and information.

During the experiment, research assistants will meticulously record all instances of adverse psychological states, the measures taken, and their effectiveness to ensure traceability. They will regularly report the occurrence of adverse psychological states and the outcomes of the interventions to the ethics committee to ensure transparency and accountability in the research process. During the recording and reporting process, participants’ privacy will be strictly protected, and no personally identifiable information will be disclosed.

### 4.9 Detailed methodology allowing for replicability

The methodology of this study was designed to ensure transparency and replicability, allowing other researchers to reproduce the experimental procedures and validate the findings. Detailed descriptions of participant recruitment, experimental design, intervention protocols, and measurement tools are provided to facilitate replication in future studies.

## 5 Data analysis

### 5.1 Descriptive statistics

We conducted descriptive statistics on the three datasets (pre-intervention, first intervention, and second intervention), calculating means, standard deviations, and other statistical parameters. The following tables and charts illustrate the trends in SCL-90 data over the three stages, clearly depicting the changes in fear, anxiety, and depression symptoms before, during, and after the interventions.

Table 3 presents a case processing summary, displaying the number of valid and missing cases under different experimental conditions. Each group of experiments consists of 52 valid cases, with 0 missing cases, indicating that the data from all participants were fully collected. The experiments are divided into two main aspects: anxiety and fear, with three trials conducted for each aspect. Each trial includes a control group and an experimental group, with all groups having consistent case numbers and percentages. This indicates that the research maintained consistency and completeness during data collection, facilitating subsequent data analysis and interpretation of results.

**Table 3:** Descriptive analysis of the experimental group and control group in three experiments.

Case processing summary							
Experimental groups		Cases					
		Valid		Missing		Total	
		*N*	Percent	*N*	Percent	*N*	Percent
1st experiment	Control group	52	100.0%	0	0.0%	52	100.0%
	Experimental group	52	100.0%	0	0.0%	52	100.0%
2nd experiment	Control group	52	100.0%	0	0.0%	52	100.0%
	Experimental group	52	100.0%	0	0.0%	52	100.0%
3rd experiment	Control group	52	100.0%	0	0.0%	52	100.0%
	Experimental group	52	100.0%	0	0.0%	52	100.0%

Many statistical tests and models (such as t-tests, ANOVA, and regression analysis) assume that the data follow a normal distribution. If the data do not meet the normality assumption, non-parametric methods may be required for analysis. Therefore, testing for normality is a crucial step in guiding researchers to select the appropriate statistical method. In this study, we conducted a normality test on the data, as shown in the figure below.

Table 4 shows the normality test results for the anxiety factor, fear factor, and overall average scores in the control and experimental groups across three experiments. The Kolmogorov–Smirnov and Shapiro–Wilk tests were used to check for normal distribution. A significance level (Sig.) below 0.05 indicates a deviation from normality.

**Table 4:** Normal distribution of experimental and control groups in three experiments.

Experiment stage	Factor	Group	Kolmogorov–Smirnov sig.	Shapiro–Wilk sig.
1st	Anxiety	Control	0.000	0.000
		Experimental	0.000	0.000
	Fear	Control	0.000	0.000
		Experimental	0.000	0.000
	Overall average	Control	0.000	0.000
		Experimental	0.000	0.000
2nd	Anxiety	Control	0.000	0.000
		Experimental	0.000	0.000
	Fear	Control	0.000	0.000
		Experimental	0.000	0.000
	Overall average	Control	0.000	0.000
		Experimental	0.001	0.000
3rd	Anxiety	Control	0.000	0.000
		Experimental	0.000	0.000
	Fear	Control	0.000	0.000
		Experimental	0.000	0.000
	Overall average	Control	0.000	0.000
		Experimental	0.000	0.000

### 5.2 Differential analysis

Due to the significant normality test result of 0.00 (*p* < 0.05), it indicates that the data distribution does not conform to a normal distribution, thus failing to meet the conditions for conducting a paired sample T-test. Therefore, we performed a non-parametric analysis using the Friedman test and obtained the ranks.

**Figure 9:**
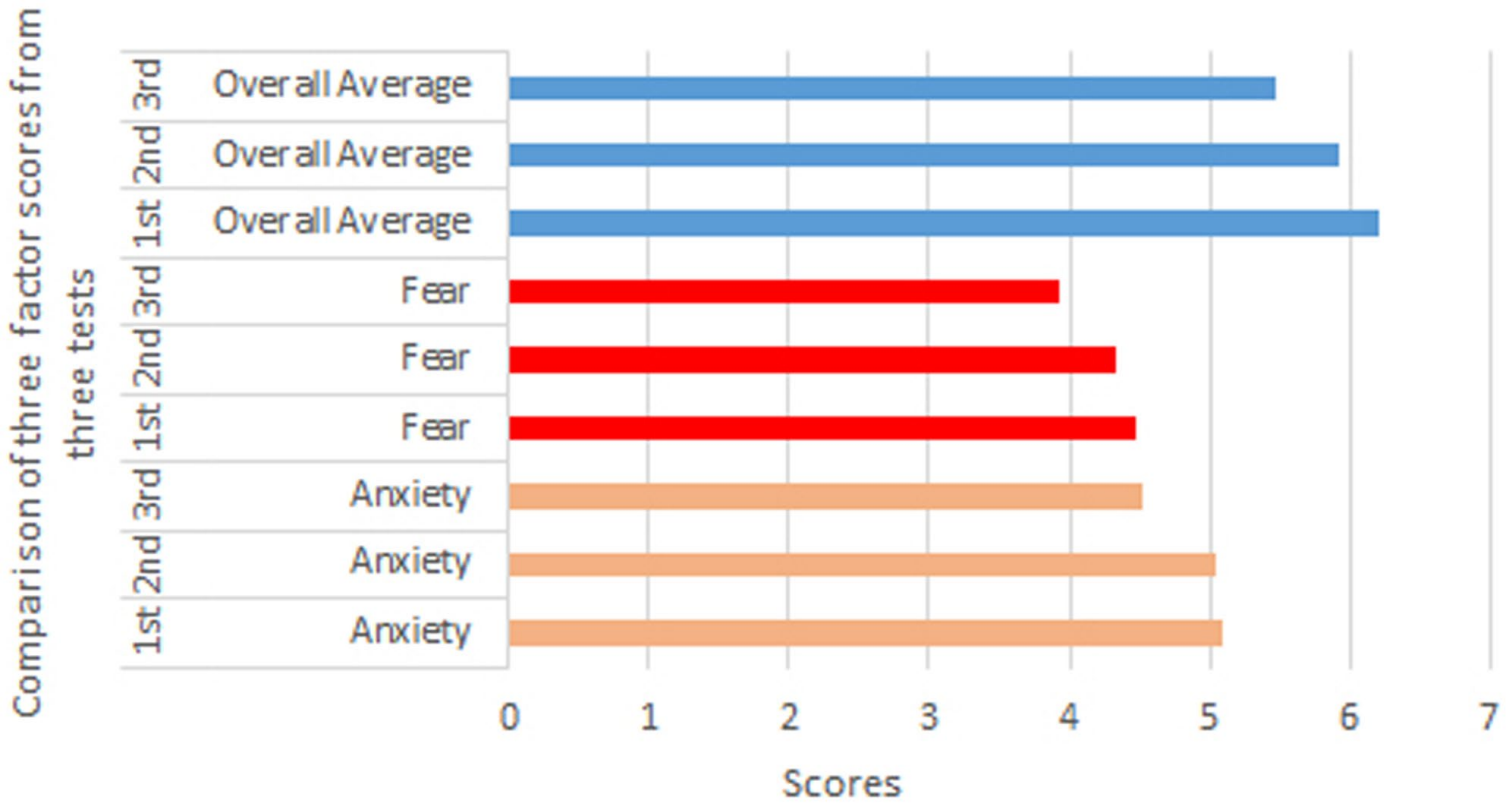
Rank mean values.

Table 5 and Figure 9 presents the rank mean values of Anxiety, Fear, and Overall Average factors across three experimental stages. The Anxiety factor shows a gradual decrease in rank mean values from 5.10 in the 1st experiment to 5.05 in the 2nd and 4.52 in the 3rd experiment, suggesting a possible reduction in anxiety levels due to the intervention. Similarly, the Fear factor’s rank mean values decrease from 4.48 in the 1st experiment to 3.93 in the 3rd, indicating a consistent downward trend. The Overall Average factor follows a similar pattern, decreasing from a rank mean of 6.21 in the 1st experiment to 5.47 in the 3rd. These trends suggest that, as the experiment progressed, participants experienced a potential reduction in anxiety and fear levels, as well as in overall symptoms, which may imply the effectiveness of the intervention and warrants further investigation.

**Table 5:** Rank mean values.

Experiment stage	Factor	Rank mean values
1st	Anxiety	5.10
2nd	Anxiety	5.05
3rd	Anxiety	4.52
1st	Fear	4.48
2nd	Fear	4.32
3rd	Fear	3.93
1st	Overall average	6.21
2nd	Overall average	5.93
3rd	Overall average	5.47

The Table 6 presents the results of the Friedman test, which was used to determine whether there are significant differences in rank values across the three time points. *N* = 104 indicates that 104 samples were included in the test. The Chi-Square value of 72.196 represents the chi-square statistic obtained from the Friedman test. The df = 8 refers to the degrees of freedom, corresponding to the number of measured variables and time points involved in the experiment. The Asymp. Sig. = 0.000 shows the asymptotic significance (*p*-value), which, being below the 0.05 threshold, indicates statistically significant differences in rank values among the time points. Table 6 suggests that the intervention led to significant changes in factors such as anxiety and fear across the three stages of the experiment. This outcome supports the potential effectiveness of the intervention in improving emotional and mental health.

**Table 6:** Friedman test statistics.

Friedman test statistics^a^	
N	104
Chi-square	72.196
df	8
Asymp. Sig.	0.000

### 5.3 Correlation analysis

We conducted a correlation analysis of the anxiety, fear, and overall average scores from the SCL-90 data collected before the experiment, after the first session, and after the second session. The tables below show the correlations between Anxiety, Fear, and Overall Average across the three time points, analyzed using Kendall’s tau-b correlation coefficients.

Table 7 shows that anxiety and fear have a correlation coefficient of 0.616, anxiety and overall average have a correlation of 0.734, and fear and overall average have a correlation of 0.717. All correlations are statistically significant at the 0.01 level (*p* = 0.000).

**Table 7:** First test correlation between anxiety, fear and overall average.

Correlations					
			1st anxiety	1st fear	1st overall average
Kendall’s tau_b	1st anxiety	Correlation coefficient	1.000	0.616^**^	0.734^**^
		Sig. (2-tailed)		0.000	0.000
		N	104	104	104
	1st fear	Correlation coefficient	0.616^**^	1.000	0.717^**^
		Sig. (2-tailed)	0.000		0.000
		N	104	104	104
	1st overall average	Correlation coefficient	0.734^**^	0.717^**^	1.000
		Sig. (2-tailed)	0.000	0.000	
		N	104	104	104

** Correlation is significant at the 0.01 level (2-tailed).

Table 8 shows that the correlation between Anxiety and Fear slightly decreases to 0.598, the correlation between Anxiety and Overall Average increases to 0.752, and the correlation between Fear and Overall Average decreases to 0.692. All correlations remain statistically significant at the 0.01 level.

**Table 8:** Second test correlation between anxiety, fear and overall average.

Correlations					
			1st anxiety	1st fear	1st overall average
Kendall’s tau_b	2nd anxiety	Correlation coefficient	1.000	0.598^**^	0.752^**^
		Sig. (2-tailed)		0.000	0.000
		N	104	104	104
	2nd fear	Correlation coefficient	0.598^**^	1.000	0.692^**^
		Sig. (2-tailed)	0.000		0.000
		N	104	104	104
	2nd overall average	Correlation coefficient	0.752^**^	0.717^**^	1.000
		Sig. (2-tailed)	0.000	0.000	
		N	104	104	104

** Correlation is significant at the 0.01 level (2-tailed).

Table 9 shows that the correlation between Anxiety and Fear increases to 0.635, the correlation between Anxiety and Overall Average strengthens to 0.776, and the correlation between Fear and Overall Average rises to 0.730. All correlations remain significant at the 0.01 level.

**Table 9:** Third test correlation between anxiety, fear and overall average.

Correlations					
			1st anxiety	1st fear	1st overall average
Kendall’s tau_b	3rd anxiety	Correlation coefficient	1.000	0.635^*^	0.776^**^
		Sig. (2-tailed)		0.000	0.000
		N	104	104	104
	3rd fear	Correlation coefficient	0.635^**^	1.000	0.730^**^
		Sig. (2-tailed)	0.000		0.000
		N	104	104	104
	3rd overall average	Correlation coefficient	0.776^**^	0.730^**^	1.000
		Sig. (2-tailed)	0.000	0.000	
		N	104	104	104

** Correlation is significant at the 0.01 level (2-tailed).

Across the three time points, significant positive correlations are observed among Anxiety, Fear, and Overall Average, with slight variations over time. Specifically, the correlation between Anxiety and Overall Average gradually strengthens, while correlations involving Fear show some fluctuation. These results indicate dynamic changes in the relationship between emotional factors and overall mental health following the intervention, supporting the effectiveness of the intervention and providing insight for further analysis.

### 5.4 Reliability and validity analysis

In this study, the reliability analysis of the anxiety factor, fear factor, and total average scores across three measurements was conducted using Cronbach’s Alpha coefficient. The results indicated that the Cronbach’s Alpha value for the scale was 0.967, demonstrating a very high level of internal consistency (see Table 1). The Cronbach’s Alpha value based on standardized items was also 0.967, further validating the consistency across different measurement scales. Additionally, this analysis involved a total of 9 items.

According to Nunnally (1978) standards, a Cronbach’s Alpha value above 0.7 is considered indicative of good internal consistency. The Alpha value in this study significantly exceeds this threshold, indicating a high degree of correlation among the selected items. This suggests that the anxiety factor, fear factor, and total average score consistently and reliably measure the same psychological constructs, confirming the reliability of the scale’s application in this study.

This Table 10 provides the reliability statistics for the measurement scale used in the study. Cronbach’s alpha, a measure of internal consistency, was calculated for the scale. The Cronbach’s alpha value of 0.967 indicates excellent reliability, as it is well above the commonly accepted threshold of 0.70. Additionally, the Cronbach’s alpha based on standardized items is also 0.967, further confirming the high consistency of the scale. The scale consisted of 9 items, which collectively demonstrated strong reliability, suggesting that the items are highly correlated and measure the same underlying construct effectively. These results support the use of this scale for the study’s purposes.

**Table 10:** Reliability statistics.

Reliability statistics		
Cronbach’s Alpha	Cronbach’s Alpha based on standardized items	*N* of items
0.967	0.967	9

In this study, the suitability of the data for factor analysis was assessed using the Kaiser-Meyer-Olkin (KMO) measure and Bartlett’s test of sphere city. The KMO measure of sampling adequacy was 0.804, indicating that the data is suitable for factor analysis. According to Kaiser’s criteria, a KMO value above 0.8 is considered a high standard for conducting factor analysis (Kaiser, 1974).

Additionally, the results of Bartlett’s test of sphericity showed a chi-square statistic of 1541.823, with 36 degrees of freedom and a significance level of 0.000 (see Table 3), which significantly rejects the null hypothesis that the correlation matrix is an identity matrix. This indicates that there is a significant correlation among the variables, making the data suitable for factor analysis.

Therefore, both the KMO measure and the results of Bartlett’s test of sphericity indicate that the data in this study are appropriate for factor analysis, providing a statistical basis for the subsequent exploration of factor structures.

### 5.5 Regression analysis

This study employed multiple linear regression analysis to explore the effects of several socio-economic variables on the dependent variable trans1. The independent variables included:

Gender (0 = female, 1 = male), Family Structure (1 = intact family, 2 = restructured family, 3 = single-parent family), Place of Origin (1 = rural, 2 = town, 3 = urban), Experience of Left-behind Status (1 = yes, 0 = no), Family Economic Status (1 = poor, 2 = average), Family Atmosphere (1 = harmonious, 2 = average, 3 = poor, 4 = unknown), this approach allows for the assessment of how these socio-economic factors influence the outcomes measured by trans1.

#### 5.5.1 Regression model

The summary of the regression model is as follows:

R-squared: 0.104, Adjusted R-squared: 0.098, Standard Error of the Estimate: 0.52243.

These results indicate that the selected independent variables have a low explanatory power regarding the dependent variable trans1 (Adjusted R-squared = 0.098). Even with the inclusion of multiple independent variables in the model, the extent to which the model explains the variance in the dependent variable remains limited.

#### 5.5.2 Analysis of variance (ANOVA)

The results of the analysis of variance (ANOVA) for the model indicate the following:

Regression Sum of Squares: 29.275, Residual Sum of Squares: 253.552, *F* Value: 17.877, Significance: 0.000.

The ANOVA results demonstrate that the overall regression model is significant (*p* < 0.001), indicating that the independent variables have a significant effect on the dependent variable, trans1.

The regression coefficients table provides insights into the influence of each independent variable on the dependent variable, trans1:

Family Atmosphere (Harmonious, Average, Poor, Do not Know): *β* = 0.238, t = 8.135, significance *p* < 0.001. This indicates that family atmosphere has a significant positive impact on trans1.

Family Structure (Normal, Reorganized, Single-Parent): β = −0.032, t = −1.015, significance *p* = 0.311. This variable does not significantly affect trans1.

Family Economic Status (Poor, Average): β = 0.029, t = 0.776, significance *p* = 0.438. This variable does not significantly affect trans1.

Place of Origin (Rural, Town, City): β = 0.000, t = 0.006, significance *p* = 0.995. This variable does not significantly affect trans1.

Experience of Being Left Behind: β = 0.114, t = 2.986, significance *p* = 0.003. This variable has a significant positive impact on trans1.

Gender (Female, Male): β = −0.032, t = −0.844, significance *p* = 0.399. This variable does not significantly affect trans1.

Overall, the analysis suggests that family atmosphere and the experience of being left behind are significant predictors of trans1, while family structure, economic status, place of origin, and gender do not show a significant impact.

Specifically, the analysis reveals that family atmosphere and the experience of being left behind significantly affect the dependent variable, trans1.

##### 5.5.2.1 Family atmosphere

The positive impact of family atmosphere indicates that a more harmonious family environment strengthens its positive influence on trans1. This suggests that individuals from supportive family backgrounds tend to score higher on trans1, reflecting better overall psychological well-being or adjustment.

##### 5.5.2.2 Experience of being left behind

The positive effect of having been left behind indicates that individuals with such experiences have a significant impact on their trans1 scores. This may imply that these experiences shape their psychological responses and coping mechanisms in a way that influences their overall mental health.

Conversely, other independent variables, such as family structure, economic status, place of origin, and gender, do not show significant effects on trans1. This may suggest that these factors have a limited explanatory power regarding trans1 in the current model.

Although the model demonstrates significance in explaining the dependent variable, trans1, its explanatory power is relatively low. Future research could consider incorporating additional relevant independent variables or utilizing different methodologies to enhance the model’s explanatory capacity. Furthermore, conducting in-depth studies on the significant variables may provide greater insights into the factors influencing trans1.

## 6 Results

### 6.1 Percentage change calculation

**Figure 10:**
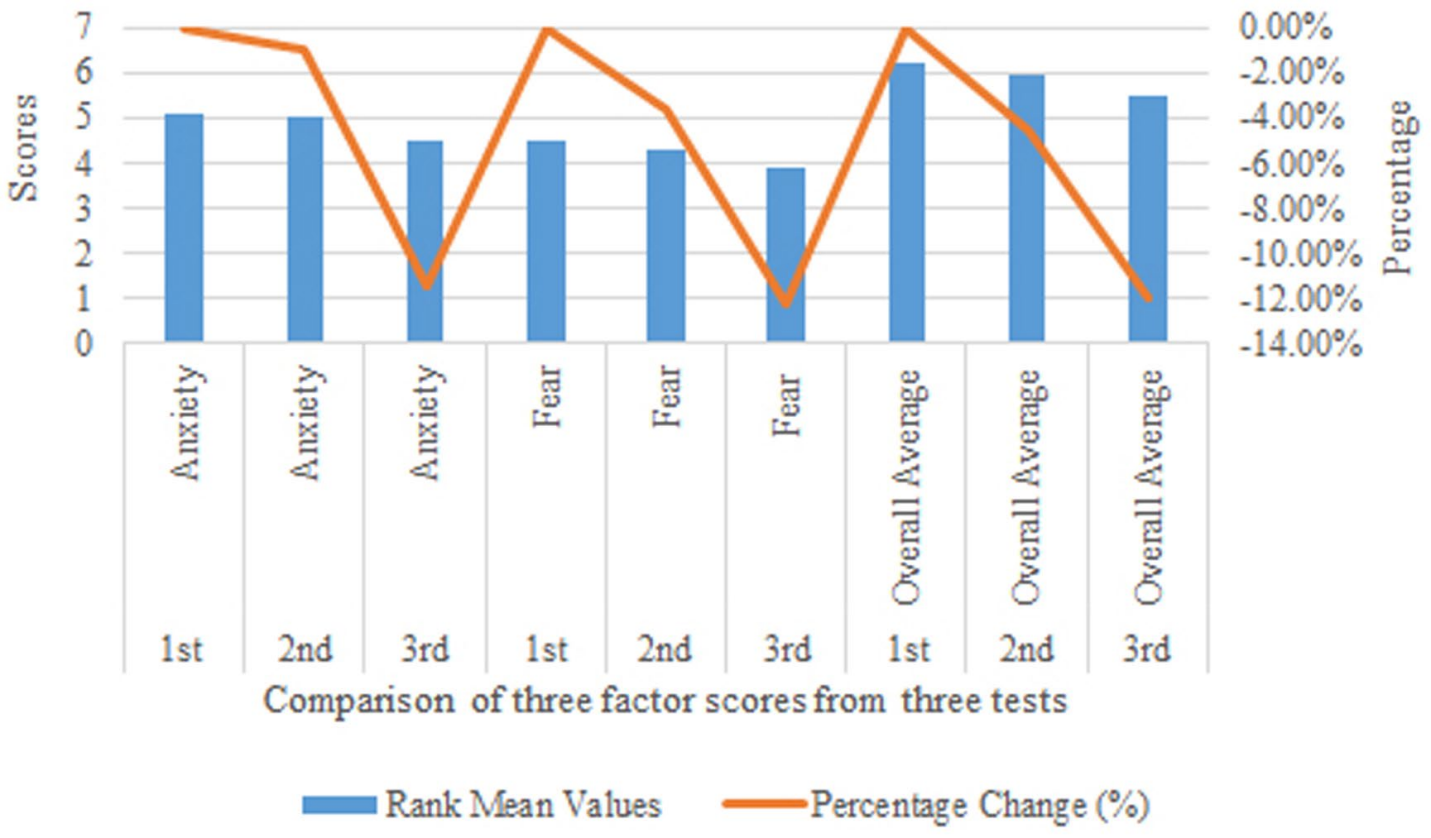
Intervention impact on anxiety and fear: rank mean values and percentage change across stages.

Based on the rank mean values from Table 5, the percentage changes in anxiety, fear, and overall average scores were calculated as follows:

Based on Table 12, select the data area containing “Experiment Stage,” “Factor,” and “Percentage Change (%)” to generate Bar Chart Figure 10. Below is a descriptive explanation of Table 12 and Figure 10.

**Table 11:** KMO and Bartlett’s test.

KMO and Bartlett’s test		
Kaiser-Meyer-Olkin measure of sampling adequacy.		0.804
Bartlett’s test of Sphericity	Approx. Chi-Square	1541.823
	df	36
	Sig.	0.000

The rank mean values for anxiety decreased progressively across the three intervention stages, from 5.1 in the pre-intervention stage to 4.52 in the post-second intervention stage. The percentage change indicates a reduction of 11.37% in anxiety scores by the end of the intervention. This suggests that the VR intervention had a significant impact on alleviating anxiety symptoms among participants (see Figure 10).

Similarly, the rank mean values for fear decreased from 4.48 in the pre-intervention stage to 3.93 in the post-second intervention stage, with a percentage change of −12.28%. This indicates a substantial reduction in fear symptoms, further supporting the effectiveness of the VR intervention in addressing fear-related psychological issues.

The overall mental health scores also showed a consistent decline, with the rank mean values decreasing from 6.21 in the pre-intervention stage to 5.47 in the post-second intervention stage. The percentage change of −11.92% reflects a significant improvement in participants’ overall psychological well-being, highlighting the comprehensive benefits of the VR intervention.

The progressive reduction in rank mean values across all three factors (anxiety, fear, and overall mental health) demonstrates the cumulative effectiveness of the VR intervention over time.

The percentage changes indicate that the most significant improvements occurred between the first and second interventions, suggesting that repeated exposure to VR scenarios enhances emotional regulation and psychological resilience.

### 6.2 Comparison of age and gender results

Participants were categorized into four groups based on gender (male and female) and age (18–20 and 21–23 years). The anxiety, fear, and total SCL-90 scores were recorded at three time points: before the first VR intervention (VR1), after the second VR intervention (VR2), and after the third VR intervention (VR3). The percentage reduction in scores was calculated by comparing the pre-intervention (VR1) and post-intervention (VR3) values for each group.

Table 13 indicates that participants and younger students (aged 18–20) showed greater improvements in anxiety and fear scores compared to male participants and older students (aged 21–23). This suggests that demographic factors, such as gender and age, may influence the effectiveness of VR interventions. These findings highlight the importance of tailoring mental health interventions to specific population groups to maximize their impact (see Tables 12, 13).

**Table 12:** Rank mean values and percentage change across intervention stages.

Experiment stage	Factor	Rank mean values	Percentage change (%)
1st	Anxiety	5.1	0.00%
2nd	Anxiety	5.05	−0.98%
3rd	Anxiety	4.52	−11.37%
1st	Fear	4.48	0.00%
2nd	Fear	4.32	−3.57%
3rd	Fear	3.93	−12.28%
1st	Overall average	6.21	0.00%
2nd	Overall average	5.93	−4.51%
3rd	Overall average	5.47	−11.92%

**Table 13:** Percentage reductions in anxiety, fear, and total scores by gender and age.

Variable	Gender	Reduction in anxiety scores (%)	Reduction in fear scores (%)	Reduction in total scores (%)
Gender	Female	16.2%	19.5%	15.3%
	Male	12.5%	15.8%	12.8%
Age	18–20	16.2%	19.5%	15.3%
	21–23	15.3%	15.8%	14.8%

### 6.3 Summary of key results

According to Table 6, the Asymp. Sig. = 0.000 indicates the asymptotic significance (*p* < 0.05). This demonstrates statistically significant differences in rank values across the three time points (pre-intervention, mid-intervention, and post-intervention). Therefore, the comparison of anxiety, fear, and overall mental health status at the pre-, mid-, and post-intervention stages is valid and meaningful.

#### 6.3.1 Correlation with demographic characteristics

To further explore the impact of demographic factors, the results were analyzed by gender and age. Female participants showed a slightly greater reduction in anxiety scores (16.2%) compared to male participants (12.5%). Similarly, younger participants (aged 18–20) exhibited a more significant decrease in fear scores (19.5%) compared to older participants (aged 21–23, 15.8%). These variations suggest that demographic factors may influence the effectiveness of VR interventions, with younger females potentially benefiting the most from the training.

#### 6.3.2 Significant relief of anxiety symptoms

Analysis of the anxiety factor revealed a progressive decrease in anxiety symptoms across the pre-intervention, post-first intervention, and post-second intervention data. The results of the Friedman test indicated that the mean rank for anxiety decreased from 5.36 after the first intervention to 5.02 after the second intervention, ultimately dropping to 4.59 after the second intervention. Statistically significant differences were observed among the three time points (*p* < 0.05). This demonstrates that the VR intervention has a significant effect on alleviating anxiety symptoms.

#### 6.3.3 Significant relief of fear symptoms

Analysis of the fear factor similarly indicated a gradual reduction in fear symptoms among the participants. The mean rank for fear decreased from 4.60 after the first intervention to 4.07 after the second intervention, ultimately reaching 3.78 following the second intervention. These results suggest that the VR intervention plays a positive role in assisting participants in overcoming fear symptoms.

#### 6.3.4 Improvement of overall mental health

The analysis of the overall mean scores indicated a significant improvement in the participants’ general psychological symptoms following the VR intervention. The mean rank for the overall score decreased from 6.39 after the first intervention to 5.79 after the second intervention, further dropping to 5.41 after the second intervention. This trend reaffirms the effectiveness of VR intervention in enhancing the overall mental health status of college students.

## 7 Discussion

### 7.1 Practical implications of VR as a low-cost intervention

The findings of this study highlight the potential of VR technology as a cost-effective intervention tool for addressing anxiety and fear among university students. Unlike traditional therapies, which often require significant time and financial resources, VR interventions can be implemented at a relatively low cost, especially with the increasing affordability of VR hardware and software through rental options. This makes VR a viable choice for educational institutions seeking to provide mental health support to students without overburdening their budgets.

### 7.2 Comparison with VR studies on specific phobias and depression

The results of this study are consistent with previous research demonstrating the efficacy of VR interventions for specific phobias, such as acrophobia and social anxiety (Witte et al., 2020). However, unlike studies focusing on specific phobias, this study addresses generalized anxiety and fear, which are more prevalent among university students. The findings suggest that VR technology can be adapted to treat a wider range of psychological issues, including those that are not strictly phobia-related.

### 7.3 Recommendations for testing VR in different settings

To further explore the potential of VR interventions, future studies should consider testing this technology in various settings, such as schools, workplaces, and community centers. For example, VR could be integrated into school counseling programs to help students manage academic stress or used in workplace wellness initiatives to reduce job-related anxiety. By expanding the contexts in which VR is applied, researchers can better understand its versatility and effectiveness across different populations and environments.

### 7.4 Limitations and future directions

While this study demonstrated the effectiveness of VR gaming in reducing anxiety and fear among university students, it is important to acknowledge the limitation of using a single type of VR scenario (high-altitude climbing). Although this scenario was chosen for its universal relevance to fear induction, future studies could explore a wider range of VR environments to address different types of psychological challenges. For example:

Virtual environments simulating social interactions, such as public speaking or group discussions, could help students overcome interpersonal anxieties.

Immersive natural environments, such as forests or beaches, could provide relaxation and stress relief, particularly for students experiencing academic burnout.

Scenarios involving problem-solving or time-sensitive tasks could help students develop coping mechanisms for stress and performance anxiety.

Additionally, future research could compare VR interventions with other emerging technologies in the mental health field, such as augmented reality (AR), biofeedback, and artificial intelligence (AI)-driven therapeutic tools. Each of these technologies offers unique advantages:

Unlike VR, which creates a fully immersive virtual environment, AR overlays digital elements onto the real world. This hybrid approach could be particularly useful for interventions that require interaction with real-world contexts, such as exposure therapy for social anxiety in public spaces.

By providing real-time physiological data (e.g., heart rate, skin conductance), biofeedback can help individuals become more aware of their stress responses and learn to regulate them. Combining biofeedback with VR could enhance the effectiveness of interventions by allowing participants to monitor their physiological reactions while engaging in virtual scenarios.

AI can personalize mental health interventions by analyzing individual psychological profiles and tailoring scenarios to specific needs. For example, AI could dynamically adjust the difficulty of VR tasks based on a participant’s progress, ensuring a more adaptive and effective therapeutic experience.

By integrating these technologies, future studies could develop more comprehensive and personalized mental health interventions, addressing a broader range of psychological issues and improving accessibility for diverse populations.

### 7.5 Practical implications for large-scale implementation in universities

The findings of this study highlight the potential of VR technology as a scalable and cost-effective intervention tool for universities. To implement VR on a large scale, institutions could consider the following strategies:

Universities could incorporate VR-based interventions into their existing mental health counseling programs. For example, VR scenarios could be used as part of group therapy sessions or individual counseling to help students practice coping strategies in a safe environment.

Establishing dedicated VR labs on campus would allow students to access VR interventions at their convenience. These labs could offer a variety of scenarios tailored to different psychological needs, such as anxiety management, stress relief, or social skills training.

Partnering with VR hardware and software providers could reduce costs and ensure access to the latest technological advancements. Universities could also explore rental or subscription models to make VR equipment more affordable.

To ensure effective implementation, university staff, including counselors and IT support teams, should receive training on how to use VR technology and integrate it into mental health programs.

Integrating biofeedback with VR could enhance interventions by providing real-time physiological data. For instance, students could use wearable devices to monitor their heart rate or stress levels while engaging in VR scenarios, allowing for more personalized and adaptive therapy.

AI-Driven Tools: AI could be used to analyze student data and tailor VR interventions to individual needs. For example, AI algorithms could adjust the difficulty of VR tasks based on a student’s progress, ensuring a more effective and engaging experience.

## 8 Limitations

While the results of this study are promising, several limitations should be acknowledged. First, the sample size of 104 participants, although sufficient for initial analysis, may limit the generalizability of the findings to broader populations. Specifically, the sample lacked diversity in terms of demographic characteristics such as age, ethnicity, and socioeconomic status, which restricts the applicability of the results to more heterogeneous groups. Future studies should prioritize recruiting larger and more diverse samples to ensure broader external validity and to explore potential variations in treatment response across different subgroups.

Second, the intervention duration of six months, while effective, may not capture the long-term effects of VR therapy. Additionally, the absence of follow-up data beyond the intervention period is a significant limitation, as it prevents us from assessing whether the observed improvements are sustained over time. Future research should incorporate extended follow-up periods to evaluate the durability of the therapeutic effects and to identify any potential late-emerging outcomes. Longitudinal studies with these considerations are essential to provide a more comprehensive understanding of the intervention’s impact.

## 9 Conclusion

The VR intervention significantly improved participants’ scores on anxiety and fear, with particularly pronounced effects observed during the second intervention. This indicates a promising application of VR technology in mental health interventions.

Results from the second intervention suggest that VR may possess a cumulative effect, where repeated interventions further enhance the alleviation of anxiety and fear. This finding highlights the potential benefits of sustained use of VR interventions.

By providing an immersive experience, VR technology creates a safe and controlled environment for university students to confront and manage their anxiety and fear. This underscores the substantial potential for VR applications in the field of mental health, particularly in areas such as mental health education, counseling, and intervention for college students.

Overall, this study provides empirical support for VR interventions as an innovative approach to mental health treatment, demonstrating their potential in improving anxiety and fear among university students. Future research could further explore the impact of different VR scenarios and intervention models on specific psychological issues to optimize intervention strategies and offer more options for mental health services.

## Data Availability

The raw data supporting the conclusions of this article will be made available by the authors, without undue reservation.
